# Supplementary material for: Enabling High Activity Catalyst Co3O4@CeO2 for Propane Catalytic Oxidation via Inverse Loading
Source: Molecules. 2023 Aug 7;28(15):5930. doi: 10.3390/molecules28155930 (PMC10421505; doi:10.3390/molecules28155930)
Supplement: Supplementary file 1 [file molecules-28-05930-s001.zip › molecules-2452086-supplementary.pdf]

Supporting information

Enabling High Activity Catalyst Co_3O_4@CeO_2 for Propane Catalytic Oxidation via Inverse Loading

Xuan Wang [1], Wei Liang [1], Changqing Lin [2], Tie Zhang [1], Jing Zhang [1], Nan Sheng [1,*], Zhaoning Song [3,*], Jie Jiang [1], Bing Sun [1] and Wei Xu [1,*]

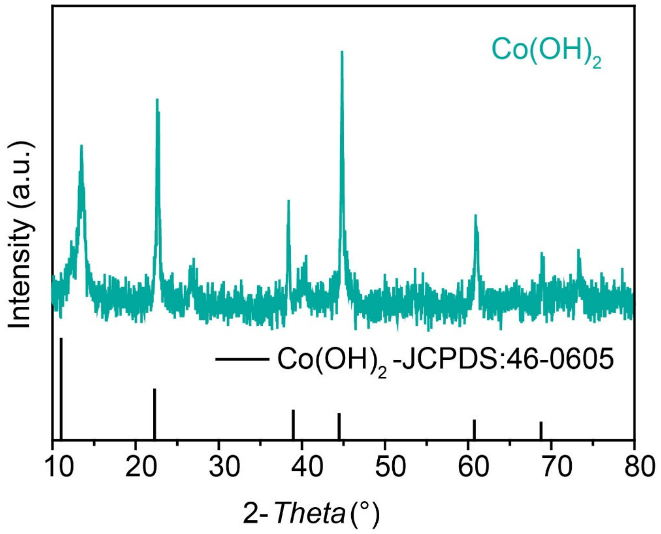

Figure. S1. PXRD patterns of $Co(OH)_2$.

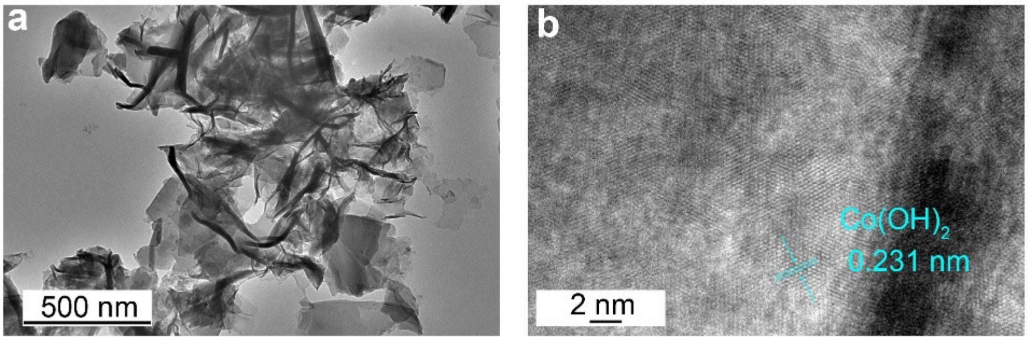

Figure. S2. Typical (a)TEM and (b) HRTEM images of $Co(OH)_2$ nanosheets.

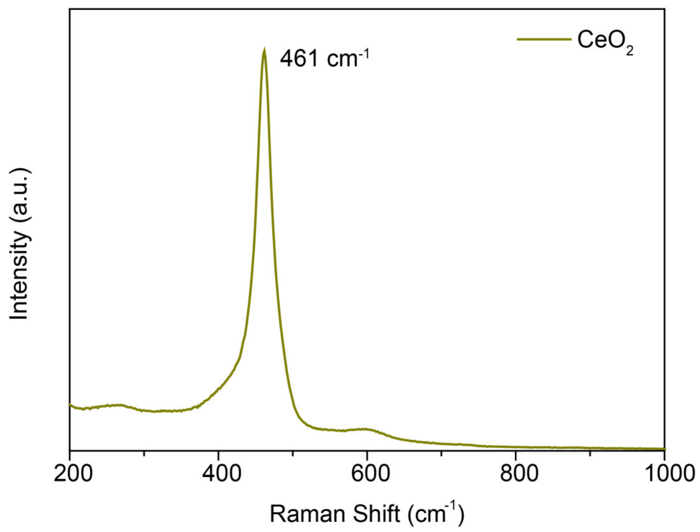

Figure. S3. Raman spectrum of CeO_2.

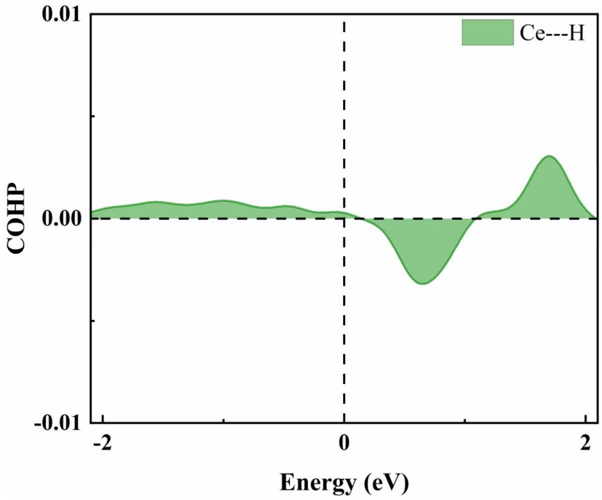

Figure. S4. The crystal orbital Hamilton population between Ce and H atoms in the Co_3O_4-OV-Ce adsorbed propane system.

Table 1 Element compositions of the two catalysts analyzed by ICP technique.

Samples	Co (at.%)	Ce (at.%)	Mg (at.%)
Co_3O_4@CeO_2-IE	45.3	8.36	1.17
Co_3O_4/CeO_2-IM	45.4	8.38	/

Table 2 . The ration of Co, Ce and O ions from XPS.

Samples	Co^{2+}/Co^{3+}(%)	Ce^{3+}/Ce^{4+}	$O_{ad}/O_V/O_L$(%)
Co_3O_4@CeO_2-IE	47.7/18.1	49.6/50.4	6/40.7/53.3

Co_3O_4/CeO_2-IM	42.1/21.5	28.6/71.4	8.4/25/66.6

Table 3. Catalytic performances of $Co_3O_4@CeO_2$-IE and Co_3O_4/CeO_2-IM catalysts.

Samples	T_{50}	T_{90}	Reaction rate[a]
$Co_3O_4@CeO_2$-IE	217	235	9.80
Co_3O_4/CeO_2-IM	268	348	6.91

[a]The feed gas was 0.5 vol% C_3H_8 and 21 vol% O_2, balanced with N_2, and T = 235 °C, GHSV = 60,000 mL h g^{-1}.
